# Association of Posttraumatic Stress Disorder With Accelerated Cognitive Decline in Middle-aged Women

**DOI:** 10.1001/jamanetworkopen.2022.17698

**Published:** 2022-06-30

**Authors:** Andrea L. Roberts, Jiaxuan Liu, Rebecca B. Lawn, Shaili C. Jha, Jennifer A. Sumner, Jae H. Kang, Eric B. Rimm, Francine Grodstein, Laura D. Kubzansky, Lori B. Chibnik, Karestan C. Koenen

**Affiliations:** 1 Department of Environmental Health, Harvard T.H. Chan School of Public Health, Boston, Massachusetts; 2 Department of Epidemiology, Harvard T.H. Chan School of Public Health, Boston, Massachusetts; 3 Department of Psychology, University of California, Los Angeles; 4 Channing Division of Network Medicine, Department of Medicine, Brigham and Women’s Hospital and Harvard Medical School, Boston, Massachusetts; 5 Department of Nutrition, Harvard T.H. Chan School of Public Health, Boston, Massachusetts; 6 Rush Alzheimer's Disease Center, Rush University Medical Center, Chicago, Illinois; 7 Department of Social and Behavioral Sciences, Harvard T.H. Chan School of Public Health, Boston, Massachusetts; 8 Department of Neurology, Massachusetts General Hospital, Boston; 9 Psychiatric and Neurodevelopmental Genetics Unit, Department of Psychiatry, Massachusetts General Hospital, Boston

## Abstract

**Importance:**

Posttraumatic stress disorder (PTSD) has been hypothesized to lead to impaired cognitive function. However, no large-scale studies have assessed whether PTSD is prospectively associated with cognitive decline in middle-aged adults.

**Objective:**

To assess the association between PTSD and decline in cognitive function over time.

**Design, Setting, and Participants:**

This cohort study included participants from the Nurses’ Health Study II, an ongoing longitudinal cohort study involving community-dwelling middle-aged female nurses residing in the US who had at least a 2-year nursing degree at the time of enrollment in 1989. The present study included 12 270 trauma-exposed women who were enrolled in the PTSD substudy of the Nurses’ Health Study II and completed 1 to 5 cognitive assessments. Data were collected from March 1, 2008, to July 30, 2019.

**Exposures:**

Lifetime PTSD symptoms, assessed using a validated questionnaire between March 1, 2008, and February 28, 2010.

**Main Outcomes and Measures:**

The main outcome was evaluated using the Cogstate Brief Battery, a self-administered online cognitive battery. Cognitive function was measured by a psychomotor speed and attention composite score and a learning and working memory composite score. Women completed the Cogstate Brief Battery every 6 or 12 months (up to 24 months) from October 3, 2014, to July 30, 2019. Linear mixed-effects models were used to evaluate the association of PTSD symptoms with the rate of change in cognition over follow-up, considering a broad range of relevant covariates, including the presence of depression symptoms and history of clinician-diagnosed depression. The rate of cognitive change was adjusted for potential practice effects (ie, potential changes in test results that occur when a test is taken more than once) by including indicators for the number of previous tests taken.

**Results:**

Among 12 270 women, the mean (SD) age at the baseline cognitive assessment was 61.1 (4.6) years; 125 women (1.0%) were Asian, 75 (0.6%) were Black, 156 (1.3%) were Hispanic, 11 767 (95.9%) were non-Hispanic White, and 147 (1.2%) were of other race and/or ethnicity. A higher number of PTSD symptoms was associated with worse cognitive trajectories. Compared with women with no PTSD symptoms, women with the highest symptom level (6-7 symptoms) had a significantly worse rate of change in both learning and working memory (β = −0.08 SD/y; 95% CI, −0.11 to −0.04 SD/y; *P* < .001) and psychomotor speed and attention (β = −0.05 SD/y; 95% CI, −0.09 to −0.01 SD/y; *P* = .02), adjusted for demographic characteristics. Associations were unchanged when additionally adjusted for behavioral factors (eg, 6-7 symptoms in the analysis of learning and working memory: β = −0.08 SD/y; 95% CI, −0.11 to −0.04 SD/y; *P* < .001) and health conditions (eg, 6-7 symptoms in the analysis of learning and working memory: β = −0.08 SD/y; 95% CI, −0.11 to −0.04 SD/y; *P* < .001) and were partially attenuated but still evident when further adjusted for practice effects (eg, 6-7 symptoms in the analysis of learning and working memory: β = −0.07 SD/y; 95% CI, −0.10 to −0.03 SD/y; *P* < .001) and comorbid depression (eg, 6-7 symptoms in the analysis of learning and working memory: β = −0.07 SD/y; 95% CI, −0.11 to −0.03 SD/y; *P* < .001).

**Conclusions and Relevance:**

In this large-scale prospective cohort study, PTSD was associated with accelerated cognitive decline in middle-aged women, suggesting that earlier cognitive screening among women with PTSD may be warranted. Given that cognitive decline is strongly associated with subsequent Alzheimer disease and related dementias, better understanding of this association may be important to promote healthy aging.

## Key Points

**Question:**

Is posttraumatic stress disorder (PTSD) associated with cognitive decline in middle-aged women?

**Findings:**

In this cohort study of 12 270 trauma-exposed middle-aged women, individuals with high levels of PTSD symptoms experienced significantly worse cognitive decline in learning and working memory as well as psychomotor speed and attention compared with those with no PTSD symptoms. These findings were not fully explained by demographic characteristics, behavioral factors, or health conditions, including comorbid depression.

**Meaning:**

This study’s findings highlight the importance of PTSD prevention and treatment to promote healthy cognitive aging and suggest that earlier cognitive screening among women with PTSD should be considered.

## Teaser

This cohort study assesses the association between posttraumatic stress disorder and cognitive decline among middle-aged women.

## Introduction

Cognitive decline at midlife and older is of substantial public health interest because it is a risk factor for worse health in a wide variety of domains. Cognitive decline has been associated with increased sedentary behavior,^1^ higher risk of hospitalization,^2^ incident frailty,^3^ and death.^4,5^ Moreover, cognitive decline has been strongly associated with subsequent Alzheimer disease and related dementias.^6,7^ Thus, identifying novel risk and protective factors associated with cognitive decline is important. Stress and posttraumatic stress disorder (PTSD) have been hypothesized to impair learning and memory by biasing attention toward threat and reducing attention to emotionally neutral information,^8,9^ altering brain structures,^10^ affecting brain immune function,^11^ and accelerating Alzheimer disease pathogenesis,^12^ with animal studies supporting the hypothesis that PTSD impairs cognitive function.^13,14^ However, although PTSD has been strongly associated with lower cognitive function in cross-sectional studies,^15,16,17,18,19^ whether PTSD is associated with subsequent decline in cognitive function is largely unknown because few longitudinal studies have been conducted. Moreover, both PTSD and dementia are more prevalent in women than in men.^20,21^ An estimated 8.6% of US women have had PTSD in their lifetime compared with 4.1% of men^22^; at age 65 years, US women have a 21.1% lifetime risk of Alzheimer disease compared with 11.6% among US men.^23^ Thus, PTSD may be an important risk factor for cognitive decline and dementia in women. However, to our knowledge, no large-scale study has examined whether PTSD is associated with decline in cognitive function among middle-aged women. Longitudinal studies have been performed among young persons,^17,18,24^ soldiers,^16,18,25^ and Holocaust survivors^26^; most of these studies have been of small or moderate size^16,18,24,25,26^ and have reported mixed findings. It remains unknown (1) whether PTSD is associated with subsequent cognitive decline; (2) the extent to which health conditions (eg, hypertension and diabetes) and behaviors (eg, smoking and alcohol consumption) are associated with both PTSD and cognitive decline^27,28^; and (3) the extent to which depression, which frequently co-occurs with PTSD^29^ and has been associated with cognitive decline,^30^ accounts for any association.

In the present cohort study, we examined PTSD symptoms and their association with repeated measures of cognitive function among a large civilian cohort of trauma-exposed women aged 50 to 71 years at study baseline. We further assessed the extent to which health conditions and behaviors might explain any observed differences in the rate of cognitive decline by PTSD status. We also examined the association of PTSD with change in cognitive function, adjusted for depressive symptoms. Our hypothesis, which was formulated before collection of data on cognitive function, was that (1) trauma-exposed women who had a high number of PTSD symptoms would have accelerated cognitive decline vs trauma-exposed women who had no PTSD symptoms and (2) health behaviors and the presence of depression and history of clinician-diagnosed depression would partly account for this difference.

## Methods

### Study Participants

The Nurses’ Health Study II comprises 116 429 US female nurses aged 25 to 42 years at enrollment in 1989. Women complete biennial questionnaires, with follow-up ongoing.^31^ In 2008, a supplemental questionnaire on trauma exposure and PTSD was mailed to 60 804 women who had returned the 2007 biennial questionnaire; 54 763 women returned this supplemental questionnaire (90% response rate). From 2014, the analytic baseline for the current cohort study, 43 957 of these women with known email addresses were invited to complete an initial cognitive assessment. A total of 15 138 women completed this assessment (34% response rate) and were invited to complete additional assessments every 6 or 12 months for up to 24 months after baseline. Of those, 12 270 trauma-exposed women were included in the current study. Women who did not respond to a cognitive assessment were not invited to complete the next assessment (eFigure 1 in the Supplement). The study was approved by the institutional review board of Brigham and Women’s Hospital. Return of questionnaires constituted implied informed consent. This study followed the Strengthening the Reporting of Observational Studies in Epidemiology (STROBE) reporting guideline for cohort studies.

### Trauma and PTSD

Lifetime trauma exposure and PTSD symptoms were assessed between March 1, 2008, and February 28, 2010. Women were asked about their exposure to 16 traumatic events (eg, physical assault and natural disaster).^32^ For women who reported trauma exposure, experience of 7 PTSD symptoms in relation to their worst trauma were assessed using the Short Screening Scale for *Diagnostic and Statistical Manual of Mental Disorders* (Fourth Edition) PTSD.^33^ In a validation study, a score of 4 or higher identified PTSD cases with sensitivity of 85% and specificity of 93%.^33^ We classified trauma-exposed women into 4 groups: (1) no PTSD symptoms, (2) 1 to 3 PTSD symptoms, (3) 4 to 5 PTSD symptoms, and (4) 6 to 7 PTSD symptoms.^34^

### Cognitive Assessment

Women completed the Cogstate Brief Battery,^35^ a validated self-administered online cognitive assessment, from October 3, 2014, to July 30, 2019. The Cogstate Brief Battery includes 4 tasks: detection (measuring psychomotor speed), identification (measuring attention), one card learning (measuring visual learning), and one back (measuring working memory).^35,36^ This instrument has been reported to have good construct and criterion validity,^37,38^ test-retest reliability,^35,39,40^ good acceptability and efficiency in studies of older individuals,^35,36^ and clinical utility in identifying cognitive impairments and dementia.^37,38,39,41^ Task scores were transformed to improve normality, with reaction times log transformed and accuracy arcsine transformed.^19,36,42^ Using established thresholds, we excluded women who did not pass integrity checks (0.90% at baseline and 0%-0.04% during follow-up). For each task, scores were standardized using means and SDs at baseline. We created 2 composite scores, with higher scores reflecting better cognitive function: (1) psychomotor speed and attention, comprising the mean of the standardized detection and identification scores, and (2) learning and working memory, comprising the mean of the standardized one card learning and one back scores. These composite scores were validated in our sample using confirmatory factor analysis^19^ and were found to be sensitive measures of cognitive decline among other samples.^39,43^

### Covariates

Covariates included factors potentially associated with cognitive decline. Demographic factors included age at baseline, self-identified race and ethnicity (Asian, Black, Hispanic, non-Hispanic White, or other), parental educational level at participant’s birth (high school or less, some college, or 4 years of college or more), and participant’s highest educational level (associate’s degree, bachelor’s degree, master’s degree, or doctoral degree). Behavior-related health factors ascertained in 2013 included body mass index (BMI; calculated as weight in kilograms divided by height in meters squared),^44^ physical activity (<3 metabolic equivalent of task [MET] hours/week, 3 to <9 MET hours/week, 9 to <18 MET hours/week, 18 to <27 MET hours/week, or ≥27 MET hours/week), and cigarette smoking (nonsmoker, former smoker, or current smoker of 1-14 cigarettes/d, 15-24 cigarettes/d, or ≥25 cigarettes/d); factors ascertained in 2011 were diet quality, measured using the Alternative Healthy Eating Index without the alcohol consumption component (score range, 0-100, with higher scores indicating better diet quality),^45^ and alcohol consumption (0 g/d, 1 to <5 g/d, 5 to <10 g/d, 10 to <20 g/d, or ≥20 g/d). Measurements closest to the baseline cognitive assessment were used. Health conditions included lifetime history (yes or no) of clinician-diagnosed hypertension, type 2 diabetes, stroke, and myocardial infarction, which were ascertained via self-report between 1989 and 2013.

Depressive symptoms over the past week were assessed in 2008 using the 10-item Center for Epidemiologic Studies Depression scale (score range, 0-30, with higher scores indicating greater severity of depressive symptoms), which has excellent psychometric properties.^46^ Clinician diagnosis of depression was self-reported from 2003 to 2013.

Fewer than 5.0% of covariates were missing, with the exception of participant educational level (24.4% missing), which was assessed through a supplemental 2018 questionnaire that was only administered in a subsample. We imputed missing values using PTSD group–specific means or modes as appropriate.

Because our exposure was lifetime PTSD measured in 2008, and many cognitive risk factors (eg, hypertension and obesity) develop over decades, it was not possible to sequence the emergence of PTSD, cognitive risk factors, and depression; thus, we could not assess whether they were confounders or mediators (eFigure 2 in the Supplement).

### Statistical Analysis

We compared 15 138 women who participated in the cognitive study with 28 819 women who declined to participate. Demographic and health characteristics were compared across PTSD symptom groups among trauma-exposed participants in the cognitive study. We further compared characteristics of trauma-exposed women who completed different numbers of cognitive assessments (range, 1-5).

We next assessed the rate of change in the cognitive composite scores using linear mixed-effects models. We accounted for within-individual associations between repeated measurements by including time since baseline as well as random intercepts and random slopes of time in all models. We evaluated statistical assumptions of linear mixed-effects models (ie, independence of residuals, homoscedasticity of residuals, and normality of residuals and random effects) and found no evidence of violation. Scores improve with repeated testing for most cognitive batteries, including the Cogstate Brief Battery.^35,47,48^ We therefore also estimated the rate of cognitive change adjusted for these potential practice effects (ie, potential changes in test results that occur when a test is taken more than once) by including indicators for the number of previous tests taken (range, 0-4).^49^

To estimate the association of PTSD with the rates of cognitive change, we included PTSD groups and their interactions with time, using trauma-exposed women with no PTSD symptoms as the reference group. We also tested for linear trend by fitting models with PTSD symptom severity level as an ordinal variable (severity level, 1-4, with 1 indicating 0 symptoms, 2 indicating 1-3 symptoms, 3 indicating 4-5 symptoms, and 4 indicating 6-7 symptoms). In the first model, we adjusted for age at baseline cognitive assessment, race and ethnicity, parental educational level, and participant educational level. To examine the consequences of behavior-related health factors for the possible association between PTSD and cognitive change, we also adjusted for BMI, smoking status, alcohol consumption, physical activity, and diet quality in the second model. Health conditions, including history of hypertension, diabetes, stroke, and myocardial infarction, were added in a third model. Covariates and covariate-time interaction terms were included for all covariates to allow any identified associations between covariates and cognition to vary across time. We also fit models restricted to women with at least 1 cognitive assessment after baseline.

We conducted 3 secondary analyses. First, because depression is a common comorbidity of PTSD,^29^ and depression has been associated with cognitive decline,^30^ we estimated the association of PTSD with cognitive changes, further adjusting for depressive symptoms (assessed in 2008) and history of depression (assessed from 2003 to 2013). Second, we examined the consequences of potential practice effects for the estimate of the association between PTSD and cognition by including indicators for the number of previous tests taken. Third, we evaluated the possibility that differential study withdrawal by PTSD symptom level across the follow-up period may have biased estimations. We fit separate linear mixed-effects models using data up to the second, third, and fourth cognitive test and compared the estimates with the main results (which included up to 5 tests).

Statistical analyses were performed using SAS software, version 9.4 (SAS Institute Inc). Hypothesis tests were 2-sided, and *P* < .05 was considered statistically significant.

## Results

Among 12 270 women included in the study, the mean (SD) age was 61.1 (4.6) years at the baseline cognitive assessment; 125 women (1.0%) were Asian, 75 (0.6%) were Black, 156 (1.3%) were Hispanic, 11 767 (95.9%) were non-Hispanic White, and 147 (1.2%) were of other race and/or ethnicity. A total of 8218 women (67.0%) reported experiencing PTSD symptoms. The distributions of worst trauma type and PTSD symptoms are shown in eTable 1 in the Supplement. Participant characteristics and baseline cognitive function by number of PTSD symptoms are shown in Table 1. A total of 4052 women had no PTSD symptoms, 5058 women had 1 to 3 symptoms, 2108 women had 4 to 5 symptoms, and 1052 women had 6 to 7 symptoms. Behavior-related health factors did not substantially differ by PTSD symptom level (eg, alcohol consumption of ≥20 g/d: 282 women [7.0%] with 0 symptoms, 405 women [8.0%] with 1-3 symptoms, 151 women [7.2%] with 4-5 symptoms, and 60 women [5.7%] with 6-7 symptoms), nor did health conditions (eg, hypertension: 1486 women [36.7%] with 0 symptoms, 1927 women [38.1%] with 1-3 symptoms, 869 women [41.2%] with 4-5 symptoms, and 436 women [41.4%] with 6-7 symptoms). Compared with women without PTSD symptoms, women with PTSD symptoms had higher depressive symptom scores (eg, 6-7 PTSD symptoms vs 0 PTSD symptoms: mean [SD], 10.3 [6.7] vs 4.4 [3.9]) and higher rates of clinician-diagnosed depression (eg, 6-7 symptoms vs 0 symptoms: 634 women [60.3%] vs 764 women [18.9%]) (Table 1).

**Table 1.** Participant Characteristics by Lifetime Number of PTSD Symptoms

Characteristic	Participants, No. (%)				
	Total (N = 12 270)	No. of PTSD symptoms			
		None (n = 4052)	1-3 (n = 5058)	4-5 (n = 2108)	6-7 (n = 1052)
Age at baseline, mean (SD), y	61.1 (4.6)	61.2 (4.6)	61.0 (4.6)	61.0 (4.5)	60.8 (4.5)
Age at worst trauma, mean (SD), y	29.7 (14.3)	28.8 (12.4)	30.2 (14.7)	30.6 (15.5)	28.9 (16.0)
Time between worst trauma and PTSD questionnaire, mean (SD), y	24.2 (14.5)	25.2 (12.8)	23.6 (14.9)	23.3 (15.5)	24.8 (16.1)
Follow-up duration, mean (SD), mo	10.6 (9.4)	10.3 (9.1)	10.7 (9.5)	10.6 (9.5)	10.9 (9.9)
Race and ethnicity^a^					
Asian	125 (1.0)	40 (1.0)	54 (1.1)	22 (1.0)	9 (0.9)
Black	75 (0.6)	26 (0.6)	34 (0.7)	11 (0.5)	4 (0.4)
Hispanic	156 (1.3)	50 (1.2)	70 (1.4)	23 (1.1)	13 (1.2)
Non-Hispanic White	11 767 (95.9)	3886 (95.9)	4850 (95.9)	2022 (95.9)	1009 (95.9)
Other	147 (1.2)	50 (1.2)	50 (1.0)	30 (1.4)	17 (1.6)
Parental educational level					
High school or less	6212 (50.6)	2107 (52.0)	2517 (49.8)	1058 (50.2)	530 (50.4)
Some college	3035 (24.7)	1011 (25.0)	1272 (25.1)	514 (24.4)	238 (22.6)
4 y of college or more	3023 (24.6)	934 (23.0)	1269 (25.1)	536 (25.4)	284 (27.0)
Participant educational level					
Associate’s degree	2302 (18.8)	867 (21.4)	834 (16.5)	398 (18.9)	203 (19.3)
Bachelor’s degree	5656 (46.1)	1807 (44.6)	2425 (47.9)	929 (44.1)	495 (47.1)
Master’s degree	3812 (31.1)	1217 (30.0)	1615 (31.9)	684 (32.4)	296 (28.1)
Doctoral degree	500 (4.1)	161 (4.0)	184 (3.6)	97 (4.6)	58 (5.5)
BMI, mean (SD)	27.5 (6.3)	27.3 (6.3)	27.3 (6.2)	27.8 (6.4)	28.0 (6.8)
Smoking status					
Never	7994 (65.2)	2704 (66.7)	3352 (66.3)	1329 (63.0)	609 (57.9)
Past	3865 (31.5)	1223 (30.2)	1554 (30.7)	701 (33.3)	387 (36.8)
Current	411 (3.3)	125 (3.1)	152 (3.0)	78 (3.7)	56 (5.3)
Alcohol consumption, g/d					
None	5143 (41.9)	1668 (41.2)	2024 (40.0)	919 (43.6)	532 (50.6)
0-20	6229 (50.8)	2102 (51.9)	2629 (52.0)	1038 (49.2)	460 (43.7)
≥20	898 (7.3)	282 (7.0)	405 (8.0)	151 (7.2)	60 (5.7)
Physical activity, mean (SD), MET h/wk	30 (30.9)	29.6 (31.0)	30.4 (30.3)	29.7 (30.8)	30.7 (34.0)
Diet quality score, mean (SD)^b^	60.2 (11.7)	59.4 (11.5)	60.4 (11.6)	60.7 (11.8)	61.3 (12.3)
Depressive symptoms score, mean (SD)^c^	6.0 (5.0)	4.4 (3.9)	5.6 (4.4)	8.1 (5.4)	10.3 (6.7)
Diagnosed depression^d^	3794 (30.9)	764 (18.9)	1409 (27.9)	987 (46.8)	634 (60.3)
Hypertension^e^	4718 (38.5)	1486 (36.7)	1927 (38.1)	869 (41.2)	436 (41.4)
Diabetes^e^	919 (7.5)	281 (6.9)	380 (7.5)	158 (7.5)	100 (9.5)
Myocardial infarction^e^	175 (1.4)	38 (0.9)	73 (1.4)	38 (1.8)	26 (2.5)
Stroke^e^	169 (1.4)	48 (1.2)	61 (1.2)	45 (2.1)	15 (1.4)
Baseline psychomotor speed and attention score, mean (SD)^f^	−0.01 (0.9)	0.03 (0.9)	−0.01 (0.9)	−0.05 (0.9)	−0.07 (1.0)
Baseline learning and working memory score, mean (SD)^f^	−0.01 (0.7)	0.01 (0.7)	0 (0.7)	−0.05 (0.7)	−0.08 (0.8)

Abbreviations: BMI, body mass index (calculated as weight in kilograms divided by height in meters squared); MET, metabolic equivalent of task; PTSD, posttraumatic stress disorder.

^a^ Choices for racial and ethnic identity included (in questionnaire order) Southern European/Mediterranean, Scandinavian, other Caucasian, which were grouped together under non-Hispanic White; African American; Hispanic; Asian; and other.

^b^ Diet quality was measured using the Alternative Healthy Eating Index without the alcohol consumption component (score range, 0-100, with higher scores indicating better diet quality).

^c^ Depressive symptoms were measured using the 10-item Center for Epidemiologic Studies Depression scale (score range, 0-30, with higher scores indicating greater severity of depressive symptoms).

^d^ History of clinician-diagnosed depression reported in the 2003-2013 questionnaires.

^e^ History of clinician-diagnosed health conditions reported in the 1989-2013 questionnaires.

^f^ Measured using standardized *z* scores, with higher scores indicating better cognitive function.

A total of 15 138 women responded to the cognitive assessment, and 28 819 did not. Responders and nonresponders were similar in demographic characteristics (eg, mean [SD] age at baseline, 53.8 [4.6] years vs 53.7 [4.6] years), health behaviors (eg, alcohol consumption of ≥20 g/d: 1119 responders [7.4%] vs 1827 nonresponders [6.3%]), health conditions (eg, hypertension: 5750 responders [38.0%] vs 11 426 nonresponders [39.7%]), and number of PTSD symptoms (eg, 6-7 symptoms: 1057 responders [7.0%] vs 2277 nonresponders [7.9%]) (eTable 2 in the Supplement). However, many differences were statistically significant because of the large size of the cohort (eg, mean [SD] age difference between responders vs nonresponders, 0.2 [4.6] years; *P* < .001).

The mean (SD) follow-up time was 0.9 (0.8) years, with 7937 women (64.7%) completing at least 2 assessments and 625 women (5.1%) completing 5 assessments. Compared with women who withdrew from the study after the baseline assessment, those who completed 5 assessments had more PTSD symptoms (eg, 6-7 symptoms: 61 of 625 women [9.8%] vs 379 of 4333 women [8.7%]) and higher scores in learning and working memory (mean [SD], 0.08 [0.71] vs −0.06 [0.75]) at baseline, but these differences were small (eTable 3 in the Supplement).

Both cognitive composite scores improved over time, likely because of practice effects. The rate of change was 0.10 SD per year (95% CI, 0.09-0.11 SD/y) in psychomotor speed and attention and 0.18 SD per year (95% CI, 0.17-0.19 SD/y) in learning and working memory, adjusted for demographic factors. After accounting for practice effects, cognitive function declined over time (psychomotor speed and attention: −0.07 SD/y [95% CI, −0.11 to −0.04 SD/y]; learning and working memory: −0.090 SD/y [95% CI, −0.12 to −0.06 SD/y]). Hereinafter, results obtained without adjustment for practice effects are referred to as rate of cognitive change, and results obtained after adjustment for practice effects are referred to as rate of cognitive decline.

### PTSD Status and Rate of Cognitive Change

A higher number of PTSD symptoms was associated with worse trajectories in both cognitive composite scores, particularly in learning and working memory, in which a strong dose-dependent association was observed (Table 2). Women with 6 to 7 PTSD symptoms had significantly worse rates of change compared with women with no symptoms (learning and working memory: β = −0.08 SD/y [95% CI, −0.11 to −0.04 SD/y; *P* < .001]; psychomotor speed and attention: β = −0.05 SD/y [95% CI, −0.09 to −0.01 SD/y; *P* = .02]), adjusted for demographic factors. Women with 4 to 5 PTSD symptoms vs no symptoms also had a significantly worse rate of change in learning and working memory (β = −0.03 SD/y; 95% CI, −0.06 to −0.003; *P* = .03) but not in psychomotor speed and attention (β = 0.01 SD/y; 95% CI, −0.02 to 0.05 SD/y; *P* = .41). Among women with 1 to 3 symptoms, changes in both cognitive composite scores were similar to those among women with no PTSD symptoms (psychomotor speed and attention: β = −0.02 SD/y [95% CI, −0.04 to 0.01 SD/y; *P* = .19]; learning and working memory: β = −0.01 SD/y [95% CI, −0.03 to 0.01 SD/y; *P* = .31]).

**Table 2.** Association Between PTSD and Rate of Change in Cogstate Composite Scores

No. of PTSD symptoms	Participants, No. (%)	Model 1^a^		Model 2^b^	
		β (95% CI), SD/y^c^	*P* value	β (95% CI), SD/y^c^	*P* value
**Psychomotor speed and attention (n = 12 248)**					
0	4044 (33.0)	[Reference]	NA	[Reference]	NA
1-3	5052 (41.2)	−0.02 (−0.04 to 0.01)	.19	−0.02 (−0.04 to 0.01)	.18
4-5	2102 (17.2)	0.01 (−0.02 to 0.05)	.41	0.01 (−0.02 to 0.05)	.42
6-7	1050 (8.6)	−0.05 (−0.09 to −0.01)	.02	−0.05 (−0.09 to −0.01)	.02
Test of trend^d^	NA	NA	.24	NA	.23
**Learning and working memory (n = 12 263)**					
No. of PTSD symptoms					
0	4050 (33.0)	[Reference]	NA	[Reference]	NA
1-3	5054 (41.2)	−0.01 (−0.03 to 0.01)	.31	−0.01 (−0.03 to 0.01)	.32
4-5	2107 (17.2)	−0.03 (−0.06 to −0.003)	.03	−0.03 (−0.06 to −0.003)	.03
6-7	1052 (8.6)	−0.08 (−0.11 to −0.04)	<.001	−0.08 (−0.11 to −0.04)	<.001
Test of trend^d^	NA	NA	<.001	NA	<.001

Abbreviations: NA, not applicable; PTSD, posttraumatic stress disorder.

^a^ Model 1 was adjusted for age at baseline cognitive assessment, race and ethnicity, parental educational level, and participant educational level.

^b^ Model 2 was adjusted for all variables in model 1 plus body mass index (calculated as weight in kilograms divided by height in meters squared), smoking status, alcohol consumption, physical activity, and diet quality.

^c^ β coefficients of the time-PTSD interaction terms from the linear mixed-effects models, representing the difference in 1-year change in Cogstate Brief Battery composite scores compared with women with no PTSD symptoms.

^d^ Linear trend was tested by fitting models with PTSD symptom severity level as an ordinal variable.

In models further adjusted for covariates and covariate-time interaction terms, the association of PTSD with cognitive change was slightly stronger (ie, further away from the null hypothesis). Several health factors were associated with lower cognitive scores: BMI (psychomotor speed and attention: β = −0.003 SD/y [95% CI, −0.006 to −0.0002 SD/y; *P* = .04]), hypertension (learning and working memory: β = −0.04 SD/y [95% CI, −0.06 to −0.01 SD/y; *P* = .01]), diabetes (psychomotor speed and attention: β = −0.12 SD/y [95% CI, −0.18 to −0.06 SD/y; *P* < .001], learning and working memory: β = −0.06 SD/y [95% CI, −0.11 to −0.01 SD/y; *P* = .01]), and stroke (learning and working memory: β = −0.18 SD/y [95% CI, −0.29 to −0.08 SD/y; *P* < .001]). However, only baseline age and myocardial infarction were significantly associated with change in learning and working memory over time (baseline age: β = −0.003 SD/y [95% CI, −0.005 to −0.0004 SD/y; *P* = .02]; myocardial infarction: β = −0.08 SD/y [95% CI, −0.16 to −0.002 SD/y; *P* = .04]). Because inclusion of covariate-time interaction terms reduced precision, findings from models adjusted for demographic factors (model 1) and further adjusted for BMI, smoking status, alcohol consumption, physical activity, and diet quality (model 2), including only covariate terms and without covariate-time interaction terms, are shown in Table 2. Results did not notably differ in the model further adjusted for history of hypertension, diabetes, stroke, and myocardial infarction. An analysis restricted to women with at least 1 cognitive assessment after baseline yielded similar results (eTable 4 in the Supplement).

### Secondary Analyses

Associations of PTSD with cognitive change were moderately attenuated when further adjusted for depressive symptoms, history of clinician-diagnosed depression, and their interactions with time. High PTSD symptom levels remained significantly associated with worse rate of change in learning and working memory in these models (eg, 6-7 symptoms in model 1: β = −0.07 SD/y [95% CI, −0.11 to −0.03 SD/y; *P* < .001]; 6-7 symptoms in model 2: β = −0.07 SD/y [95% CI, −0.11 to −0.03 SD/y; *P* < .001]) (eTable 5 in the Supplement).

After accounting for practice effects, women exhibited cognitive decline across all levels of PTSD symptoms (Figure). Associations between PTSD and cognitive change were slightly attenuated in models adjusted for practice effects (eTable 6 in the Supplement). For learning and working memory, women with 6 to 7 symptoms experienced cognitive decline approximately 2 times faster than women with no PTSD symptoms (β = −0.14 SD/y [95% CI, −0.18 to −0.09 SD/y] vs −0.07 SD/y [95% CI, −0.11 to −0.04 SD/y]; *P* < .001; equivalent to 0.69 SD/5y vs 0.36 SD/5y) (Figure). We also observed greater cognitive decline in psychomotor speed and attention among women with 6 to 7 symptoms compared with the reference group of women with no symptoms (β = −0.10 SD/y [95% CI, −0.15 to −0.05 SD/y] vs −0.07 SD/y [95% CI, −0.11 to −0.03 SD/y]; *P* = .07; equivalent to 0.51 SD/5y vs 0.33 SD/5y), although this difference was not statistically significant (eTable 6 in the Supplement).

**Figure.**
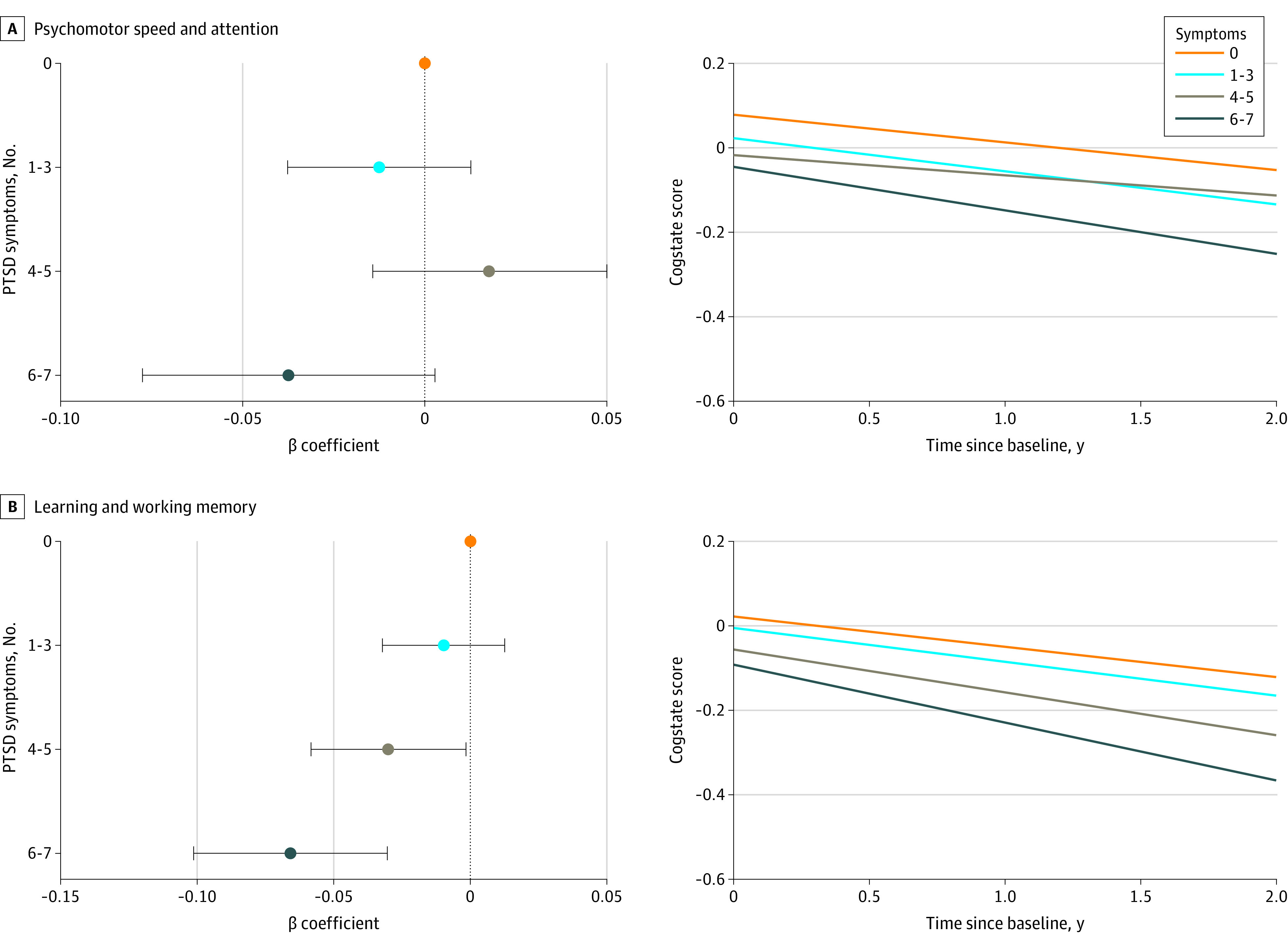
Association of PTSD With Rate of Cognitive Change and Fitted Linear Trajectories by Posttraumatic Stress Disorder (PTSD) Group The β coefficients are from models adjusted for age at baseline cognitive assessment, race and ethnicity, parental educational level, participant educational level, and number of previous cognitive tests. Error bars represent 95% CIs. Fitted linear trajectories were calculated for non-Hispanic White women (the largest race and ethnicity group in this sample) who were aged 61 years at baseline, with the highest participant educational level of bachelor’s degree and the highest parental educational level of high school.

In the analysis evaluating the consequences of differential study withdrawal across PTSD groups, we found a stronger association between 6 to 7 PTSD symptoms and cognition when we restricted follow-up to the second assessment (psychomotor speed and attention: β = −0.08 SD/y [95% CI, −0.15 to −0.01 SD/y; *P* = .03]; learning and working memory: β = −0.10 SD/y (95% CI, −0.17 to −0.04 SD/y; *P* = .001]). In the analyses restricting follow-up to the third and fourth assessments, associations were similar to estimates using all assessments (eTable 7 in the Supplement).

## Discussion

In this large longitudinal cohort study of trauma-exposed women aged 50 to 71 years, women with a high number of PTSD symptoms experienced significantly more decline in cognitive function across time than women with no PTSD symptoms. Among women with a moderate number of PTSD symptoms (ie, 4-5 symptoms), we found significantly greater cognitive decline in learning and working memory compared with women with no PTSD symptoms. In models adjusted for practice effects, women with high PTSD symptom levels had approximately 2 times faster cognitive decline in learning and working memory than those with no PTSD symptoms. Women with the highest number of PTSD symptoms experienced cognitive decline at a pace equivalent to 0.69 SD per 5 years compared with 0.36 SD per 5 years among women with no PTSD symptoms. For psychomotor speed and attention, women with the highest number of PTSD symptoms experienced cognitive decline at a pace equivalent to 0.51 SD per 5 years compared with 0.33 SD per 5 years among women with no PTSD symptoms.

At study baseline, the prevalence of cognition-related health factors did not notably differ by PTSD status. Only depressive symptoms and prevalence of clinician-diagnosed depression were substantially higher in women with vs without PTSD symptoms. Adjustment for health factors had few consequences for the association of PTSD with cognitive change, both because these factors did not differ by PTSD symptom level and because, with the exception of age and myocardial infarction, these health factors were not associated with cognitive change in our data.

Our findings were consistent with those of previous cross-sectional studies among persons exposed to extreme traumas, including military combat, the Holocaust, and childhood sexual abuse.^9,50,51,52,53^ These studies have generally found that persons with PTSD have lower cognitive function than those without PTSD.^9,50,51,52,53^ Moreover, as in our findings, the largest cognitive differences were observed in learning and memory.^53^

Our results may also inform previous findings revealing that persons with vs without PTSD have a higher risk of developing dementia.^54,55,56,57,58,59^ A previous study^60^ suggested that the risk of dementia may be higher among persons with PTSD because PTSD leads to impaired cognitive function, which results in lower cognitive reserve. In addition, lower educational level and lower socioeconomic status among persons with PTSD could be associated with a higher risk of dementia.^60,61,62^ Our study added to these findings by suggesting that PTSD may accelerate cognitive decline at midlife and older, thereby increasing the risk of dementia based on the assumption that acceleration in cognitive decline is associated with increased risk of dementia.^6,7,63^

Given the high lifetime prevalence of PTSD and dementia among women,^64^ identifying PTSD as a risk factor suggests that PTSD is not only a problem in its own right but may have implications for cognitive health. Such findings support the value of earlier cognitive screening among individuals with PTSD. Additional work may seek to identify mechanisms underlying these associations, given that established risk factors for cognitive decline (eg, age and stroke) did not account for our findings. Future work is warranted to examine whether remission of PTSD is associated with reductions in cognitive decline. More broadly, PTSD has been associated with an increased risk of several diseases and conditions of aging, including hypertension,^65^ inflammation,^66,67^ cardiovascular disease,^68,69^ type 2 diabetes,^34^ and ovarian cancer.^70^ Together, these findings suggest that PTSD prevention and treatment across the life span may improve not only mental health but also physical health and healthy aging.

### Limitations

This study has several limitations. First, our data are from an occupational cohort comprising mostly non-Hispanic White female nurses; therefore, our results may not be generalizable to other populations. Second, PTSD was assessed by a self-report screening instrument, which, though validated against a diagnostic interview, may result in misclassification. Third, we examined only lifetime PTSD symptoms. Evidence suggests that retrospective lifetime assessments of psychopathology are associated with underestimates of disorder,^71^ which may bias the associations to the null. In addition, the ways in which chronicity, timing, and remission of PTSD may be associated with cognitive decline warrants further investigation. Fourth, PTSD and cognitive decline may share risk factors.^72^ Thus, although cognitive decline occurred after the onset of PTSD in our study, unmeasured shared factors (eg, genetic or childhood factors) that occurred before the onset of PTSD and cognitive decline may have accounted for some or all of the association we found. Fifth, a substantial number of participants in our study were unavailable for follow-up during data collection. However, in a sensitivity analysis, we observed stronger associations when using shorter compared with longer follow-up times, which suggests the associations we observed may be biased toward the null, and the true associations might be stronger. Sixth, our maximum follow-up time was only 24 months. Future research might investigate the association of PTSD with cognitive decline over a longer period.

## Conclusions

This cohort study found that PTSD was associated with accelerated cognitive decline in middle-aged women. Given that cognitive decline is associated with subsequent Alzheimer disease and related dementias, better understanding of this association may be important to promote healthy aging. These findings also highlight the importance of PTSD prevention and treatment to ensure heathy cognitive aging and suggest the value of earlier cognitive screening among women with PTSD.

## Notes

Supplement. **eTable 1.** Distribution of Worst Trauma Type and PTSD Symptoms in Cogstate Sample **eTable 2.** Characteristics of Responders and Nonresponders to Cogstate Invitation **eTable 3.** Participant Characteristics by Number of Completed Cognitive Assessments **eTable 4.** Association Between PTSD and Rate of Change in Cogstate Composite Scores Among Women With at Least 1 Cognitive Assessment After Baseline **eTable 5.** Association Between PTSD and Rate of Change in Cogstate Composite Scores Adjusted for Depression **eTable 6.** Association Between PTSD and Rate of Change in Cogstate Composite Scores Adjusted for Practice Effects **eTable 7.** Association Between PTSD and Rate of Change in Cogstate Composite Scores Using Data Up to the Second, Third, and Fourth Session **eFigure 1.** Flowchart of Analytic Sample Selection **eFigure 2.** Conceptual Models of PTSD, Cognitive Decline, Depression, and Covariates
