# Supplementary material for: Association of Posttraumatic Stress Disorder With Accelerated Cognitive Decline in Middle-aged Women
Source: JAMA Netw Open. 2022 Jun 30;5(6):e2217698. doi: 10.1001/jamanetworkopen.2022.17698 (PMC9247738; doi:10.1001/jamanetworkopen.2022.17698)
Supplement: Supplement. — eTable 1. Distribution of Worst Trauma Type and PTSD Symptoms in Cogstate Sample eTable 2. Characteristics of Responders and Nonresponders to Cogstate Invitation eTable 3. Participant Characteristics by Number of Completed Cognitive Assessments eTable 4. Association Between PTSD and Rate of Change in Cogstate Composite Scores Among Women With at Least 1 Cognitive Assessment After Baseline eTable 5. Association Between PTSD and Rate of Change in Cogstate Composite Scores Adjusted for Depression eTable 6. Association Between PTSD and Rate of Change in Cogstate Composite Scores Adjusted for Practice Effects eTable 7. Association Between PTSD and Rate of Change in Cogstate Composite Scores Using Data Up to the Second, Third, and Fourth Session eFigure 1. Flowchart of Analytic Sample Selection eFigure 2. Conceptual Models of PTSD, Cognitive Decline, Depression, and Covariates [file jamanetwopen-e2217698-s001.pdf]

Supplementary Online Content

Roberts AL, Liu J, Lawn RB, et al. Association of posttraumatic stress disorder with accelerated cognitive decline in middle-aged women. *JAMA Netw Open*. 2022;5(6):e2217698. doi:10.1001/jamanetworkopen.2022.17698

eTable 1. Distribution of Worst Trauma Type and PTSD Symptoms in Cogstate Sample

eTable 2. Characteristics of Responders and Nonresponders to Cogstate Invitation

eTable 3. Participant Characteristics by Number of Completed Cognitive Assessments

eTable 4. Association Between PTSD and Rate of Change in Cogstate Composite Scores Among Women With at Least 1 Cognitive Assessment After Baseline

eTable 5. Association Between PTSD and Rate of Change in Cogstate Composite Scores Adjusted for Depression

eTable 6. Association Between PTSD and Rate of Change in Cogstate Composite Scores Adjusted for Practice Effects

eTable 7. Association Between PTSD and Rate of Change in Cogstate Composite Scores Using Data Up to the Second, Third, and Fourth Session

eFigure 1. Flowchart of Analytic Sample Selection

eFigure 2. Conceptual Models of PTSD, Cognitive Decline, Depression, and Covariates

This supplementary material has been provided by the authors to give readers additional information about their work.

eTable 1. Distribution of Worst Trauma Type and PTSD Symptoms in Cogstate Sample

	**No. (%)**
**Worst trauma type**	
Serious accident	732 (6.0%)
Natural/human-made disaster	409 (3.3%)
Life-threatening illness	802 (6.5%)
Physical punishments/beatings before age 18	550 (4.5%)
Other physical attack	645 (5.3%)
Unwanted sexual contact	1357 (11.1%)
Miscarriage/Stillbirth	1331 (10.9%)
Death of offspring	341 (2.8%)
Complications of pregnancy/labor	520 (4.2%)
Sexual harassment at work	156 (1.3%)
Serious injury or fear of injury	404 (3.3%)
Death of a close family member or friend	1626 (13.3%)
Witness of injury/death outside of work	515 (4.2%)
War-related casualties	65 (0.5%)
Treating civilians with traumatic injuries	446 (3.6%)
Other	1640 (13.4%)
**PTSD symptoms**	
Avoided being reminded of this experience by staying away from certain places, people or activities	5063 (41.3%)
Lost interest in activities that were once important or enjoyable	3615 (29.5%)
Felt more isolated or distant from other people	4771 (38.9%)
Found it hard to have love or affection for other people	2720 (22.2%)
Felt that there was no point in planning for the future	1741 (14.2%)
Had more trouble than usual falling asleep or staying asleep	4999 (40.7%)
Became jumpy or got easily startled by ordinary noises or movements	2660 (21.7%)

© 2022 Roberts AL et al. *JAMA Network Open*.

eTable 2. Characteristics of Responders and Nonresponders to Cogstate Invitation

	Responders	Non-responders	*p* [e]
	(n=15,138)	(n=28,819)	
**Age at trauma/PTSD assessment (year), mean (SD)**	53.8 (4.6)	53.7 (4.6)	<0.001
**Age at worst trauma (year), mean (SD)**	29.7 (14.3)	30.2 (14.2)	0.002
**Time between worst trauma and PTSD questionnaire (year), mean (SD)**	24.2 (14.5)	23.5 (14.4)	<0.001
**Non-Hispanic White, No. (%)**	14515 (95.9)	27346 (94.9)	<0.001
**Parental education, No. (%)**			<0.001
High school	7703 (50.9)	15348 (53.3)	
Some college	3692 (24.4)	6802 (23.6)	
College plus	3738 (24.7)	6661 (23.1)	
**Participant education, No. (%)**			<0.001
Associate’s	2881 (19.0)	4679 (16.2)	
Bachelor’s	7058 (46.6)	15016 (52.1)	
Master’s	4601 (30.4)	8374 (29.1)	
Doctorate	593 (3.9)	742 (2.6)	
**Body mass index (kg/m^2), mean (SD)**	27.4 (6.3)	27.4 (6.2)	0.39
**Smoking status, No. (%)**			<0.001
Never	9964 (65.8)	19046 (66.1)	
Past	4673 (30.9)	8529 (29.6)	
Current	496 (3.3)	1236 (4.3)	
**Alcohol intake, No. (%)**			<0.001
None	6259 (41.4)	13349 (46.3)	
0-20 grams/day	7755 (51.2)	13635 (47.3)	
20+ grams/day	1119 (7.4)	1827 (6.3)	
**Physical activity (MET-hrs/wk), mean (SD)**	30 (31.2)	28.9 (31.2)	<0.001
**Diet quality (AHEI) [a], mean (SD)**	60.1 (11.7)	59.6 (11.1)	<0.001

© 2022 Roberts AL et al. *JAMA Network Open*.

	Responders	Non-responders	*p* [e]
	(n=15,138)	(n=28,819)	
**Depressive symptoms (CES-D) [b], mean (SD)**	5.8 (4.8)	6.0 (5.0)	<0.001
**Diagnosed depression [c], No. (%)**	4382 (29.0)	8125 (28.2)	0.10
**Trauma/PTSD, No. (%)**			<0.001
No trauma	2748 (18.2)	5589 (19.4)	
Trauma no PTSD symptoms	4097 (27.1)	7871 (27.3)	
Trauma 1-3 PTSD symptoms	5104 (33.7)	9116 (31.6)	
Trauma 4-5 PTSD symptoms	2127 (14.1)	3958 (13.7)	
Trauma 6-7 PTSD symptoms	1057 (7.0)	2277 (7.9)	
**Hypertension [d], No. (%)**	5750 (38.0)	11426 (39.7)	<0.001
**Diabetes [d], No. (%)**	1093 (7.2)	2543 (8.8)	<0.001
**Myocardial infarction [d], No. (%)**	191 (1.3)	415 (1.4)	0.13
**Stroke [d], No. (%)**	190 (1.3)	465 (1.6)	0.003

Values are means (SD) for continuous variables or No. (%) for categorical variables. MET = metabolic equivalent of task.
[a] Alternate Healthy Eating Index (AHEI) without alcohol consumption was used in the analysis; higher scores reflect better diet quality (possible range=0–100).
[b] Center for Epidemiologic Studies Depression (CES-D) scale, short form (possible range=0–30).
[c] History of clinician-diagnosed depression reported at the 2003-2013 questionnaires.
[d] History of clinician-diagnosed health conditions reported at the 1989-2013 questionnaires.
[e] *P* values were obtained from two-sample t test for continuous characteristics and Chi-squared test for categorical characteristics.

eTable 3. Participant Characteristics by Number of Completed Cognitive Assessments

Number of Assessments						
	**1**	**2**	**3**	**4**	**5**	***p*** [e]
	(n=4,333)	(n=3,727)	(n=2,557)	(n=1,028)	(n=625)	
**Age at baseline (year), mean (SD)**	60.8 (4.5)	61.1 (4.5)	61.2 (4.5)	61.3 (4.7)	61.4 (4.6)	<0.001
**Age at worst trauma (year), mean (SD)**	29.6 (14.1)	29.4 (14.3)	30.3 (14.3)	29.8 (14.9)	29.4 (14.9)	0.16
**Time between worst trauma and PTSD questionnaire (year), mean (SD)**	23.9 (14.3)	24.5 (14.6)	23.8 (14.5)	24.3 (14.8)	25.1 (15)	0.14
**Non-Hispanic White, No. (%)**	4142 (95.6)	3567 (95.7)	2457 (96.1)	996 (96.9)	605 (96.8)	0.24
**Parental education, No. (%)**						0.34
High school	2133 (49.2)	1889 (50.7)	1335 (52.2)	531 (51.7)	324 (51.8)	
Some college	1106 (25.5)	908 (24.4)	629 (24.6)	250 (24.3)	142 (22.7)	
College plus	1094 (25.2)	930 (25.0)	593 (23.2)	247 (24.0)	159 (25.4)	
**Participant education, No. (%)**						<0.001
Associate’s	706 (16.3)	721 (19.3)	517 (20.2)	220 (21.4)	138 (22.1)	
Bachelor’s	2058 (47.5)	1693 (45.4)	1169 (45.7)	463 (45.0)	273 (43.7)	
Master’s	1406 (32.4)	1156 (31.0)	756 (29.6)	305 (29.7)	189 (30.2)	
Doctorate	163 (3.8)	157 (4.2)	115 (4.5)	40 (3.9)	25 (4.0)	
**Body mass index (kg/m^2), mean (SD)**	27.6 (6.2)	27.4 (6.3)	27.2 (6.2)	27.7 (6.8)	27.0 (6.1)	0.04
**Smoking status, No. (%)**						0.60

© 2022 Roberts AL et al. *JAMA Network Open*.

	Number of Assessments					
	1	2	3	4	5	*p* [e]
	(n=4,333)	(n=3,727)	(n=2,557)	(n=1,028)	(n=625)	
Never	2829 (65.3)	2422 (65.0)	1653 (64.6)	690 (67.1)	400 (64.0)	
Past	1344 (31.0)	1180 (31.7)	823 (32.2)	311 (30.3)	207 (33.1)	
Current	160 (3.7)	125 (3.4)	81 (3.2)	27 (2.6)	18 (2.9)	
**Alcohol intake, No. (%)**						0.34
None	1851 (42.7)	1515 (40.6)	1049 (41.0)	452 (44.0)	276 (44.2)	
0-20 grams/day	2163 (49.9)	1946 (52.2)	1309 (51.2)	507 (49.3)	304 (48.6)	
20+ grams/day	319 (7.4)	266 (7.1)	199 (7.8)	69 (6.7)	45 (7.2)	
**Physical activity (MET-hrs/wk), mean (SD)**	30.2 (32.2)	29.9 (30.3)	30 (31.4)	29.3 (28.1)	30.8 (28.6)	0.89
**Diet quality (AHEI) [a], mean (SD)**	60.2 (11.7)	60.3 (11.7)	60.1 (11.5)	60.0 (11.6)	60.4 (12.3)	0.90
**Depressive symptoms (CES-D) [b], mean (SD)**	6.1 (5)	6.1 (5)	5.9 (4.9)	6.1 (5)	5.9 (4.8)	0.77
**Diagnosed depression [c], %(n)**	1371 (31.6)	1142 (30.6)	774 (30.3)	331 (32.2)	176 (28.2)	0.32
**PTSD status, %(n)**						0.29
No PTSD	1429 (33.0)	1284 (34.5)	835 (32.7)	330 (32.1)	174 (27.8)	
1-3 PTSD sx	1774 (40.9)	1499 (40.2)	1073 (42.0)	439 (42.7)	273 (43.7)	
4-5 PTSD sx	751 (17.3)	623 (16.7)	443 (17.3)	174 (16.9)	117 (18.7)	
6-7 PTSD sx	379 (8.7)	321 (8.6)	206 (8.1)	85 (8.3)	61 (9.8)	
**Hypertension [d], No. (%)**	1718 (39.6)	1419 (38.1)	937 (36.6)	416 (40.5)	228 (36.5)	0.06
**Diabetes [d], No. (%)**	355 (8.2)	273 (7.3)	178 (7.0)	73 (7.1)	40 (6.4)	0.23

© 2022 Roberts AL et al. *JAMA Network Open*.

Number of Assessments						
	**1**	**2**	**3**	**4**	**5**	***p*** [e]
	(n=4,333)	(n=3,727)	(n=2,557)	(n=1,028)	(n=625)	
**Myocardial infarction [d], No. (%)**	48 (1.1)	53 (1.4)	53 (2.1)	13 (1.3)	8 (1.3)	0.03
**Stroke [d], No. (%)**	67 (1.5)	40 (1.1)	37 (1.4)	15 (1.5)	10 (1.6)	0.43
**Baseline psychomotor speed/attention [f], mean (SD)**	-0.02 (0.94)	-0.01 (0.91)	0.02 (0.88)	0.01 (0.90)	-0.02 (0.95)	0.59
**Baseline learning/working memory [f], mean (SD)**	-0.06 (0.75)	-0.00 (0.73)	0.03 (0.72)	0.02 (0.69)	0.08 (0.71)	<0.001

Values are means (SD) for continuous variables or No. (%) for categorical variables. MET = metabolic equivalent of task. sx =symptoms.
[a] Alternate Healthy Eating Index (AHEI) without alcohol consumption was used in the analysis; higher scores reflect better diet quality (possible range=0–100).
[b] Center for Epidemiologic Studies Depression (CES-D) scale, short form (possible range=0–30).
[c] History of clinician-diagnosed depression reported at the 2003-2013 questionnaires.
[d] History of clinician-diagnosed health conditions reported at the 1989-2013 questionnaires.
[e] *P* values were obtained from F test for continuous characteristics and Chi-squared test for categorical characteristics.
[f] Standardized z scores with higher values indicating better cognitive function.

© 2022 Roberts AL et al. *JAMA Network Open*.

eTable 4. Association Between PTSD and Rate of Change in Cogstate Composite Scores Among Women With at Least 1 Cognitive Assessment After Baseline

	No. (%)	Model-1		Model-2	
		b **(95% CI)[a]**	***p***	***b*** **(95% CI)[a]**	***p***
**Psychomotor Speed/Attention (n=7,895)**					
No PTSD symptoms	2,608 (33.0)	Ref	Ref	Ref	Ref
1-3 PTSD symptoms	3,266 (41.4)	-0.024 (-0.049, 0.002)	0.07	-0.024 (-0.050, 0.002)	0.07
4-5 PTSD symptoms	1,350 (17.1)	0.013 (-0.020, 0.046)	0.44	0.013 (-0.020, 0.046)	0.44
6-7 PTSD symptoms	671 (8.5)	-0.054 (-0.095, -0.013)	0.01	-0.054 (-0.095, -0.013)	0.01
**Learning/Working Memory (n=7,932)**					
No PTSD symptoms	2,608 (33.0)	Ref	Ref	Ref	Ref
1-3 PTSD symptoms	3,266 (41.4)	-0.007 (-0.030, 0.016)	0.55	-0.007 (-0.030, 0.016)	0.54
4-5 PTSD symptoms	1,350 (17.1)	-0.026 (-0.056, 0.003)	0.08	-0.026 (-0.056, 0.003)	0.08
6-7 PTSD symptoms	671 (8.5)	-0.081 (-0.118, -0.044)	<0.001	-0.082 (-0.119, -0.045)	<0.001

[a] Beta coefficients of the time x PTSD interaction terms from the linear mixed effect models are shown, representing the difference in one-year change in Cogstate composite scores compared to women with no PTSD symptoms.
Model 1: age at baseline cognitive assessment, race/ethnicity, parental education, participant education
Model 2: Model 1 + body mass index, smoking status, alcohol use, physical activity, diet quality.

© 2022 Roberts AL et al. *JAMA Network Open*.

eTable 5. Association Between PTSD and Rate of Change in Cogstate Composite Scores Adjusted for Depression

	No. (%)	Model-1		Model-2	
		***b* (95% CI)[a]**	***p***	***b* (95% CI)[a]**	***p***
**Psychomotor Speed/Attention (n=12,248)**					
No PTSD symptoms	4,044 (33.0)	Ref	Ref	Ref	Ref
1-3 PTSD symptoms	5,052 (41.3)	-0.015 (-0.040, 0.011)	0.25	-0.015 (-0.040, 0.011)	0.25
4-5 PTSD symptoms	2,102 (17.2)	0.020 (-0.013, 0.054)	0.23	0.020 (-0.013, 0.054)	0.24
6-7 PTSD symptoms	1,050 (8.6)	-0.037 (-0.080, 0.006)	0.09	-0.037 (-0.080, 0.006)	0.09
**Learning/Working Memory (n=12,263)**					
No PTSD symptoms	4,050 (33.0)	Ref	Ref	Ref	Ref
1-3 PTSD symptoms	5,054 (41.2)	-0.010 (-0.033, 0.012)	0.37	-0.010 (-0.033, 0.013)	0.38
4-5 PTSD symptoms	2,107 (17.2)	-0.028 (-0.058, 0.002)	0.07	-0.028 (-0.058, 0.002)	0.07
6-7 PTSD symptoms	1,052 (8.6)	-0.072 (-0.110, -0.033)	<0.001	-0.072 (-0.11, -0.033)	<0.001

[a] Beta coefficients of the time x PTSD interaction terms from the linear mixed effect models are shown, representing the difference in one-year change in Cogstate composite scores compared to women with no PTSD symptoms.
Model 1: age at baseline cognitive assessment, race/ethnicity, parental education, participant education, CES-D score and its interaction with time, history of clinician-diagnosed depression and its interaction with time.
Model 2: Model 1 + body mass index, smoking status, alcohol use, physical activity, diet quality.

© 2022 Roberts AL et al. *JAMA Network Open*.

eTable 6. Association Between PTSD and Rate of Change in Cogstate Composite Scores Adjusted for Practice Effects

	No. (%)	Model-1		Model-2	
		b **(95% CI)[a]**	***p***	***b*** **(95% CI)[a]**	***p***
**Psychomotor Speed/Attention (n=12,248)**					
No PTSD symptoms	4,044 (33.0)	Ref	Ref	Ref	Ref
1-3 PTSD symptoms	5,052 (41.3)	-0.012 (-0.038, 0.013)	0.33	-0.013 (-0.038, 0.013)	0.33
4-5 PTSD symptoms	2,102 (17.2)	0.018 (-0.014, 0.050)	0.28	0.018 (-0.014, 0.050)	0.28
6-7 PTSD symptoms	1,050 (8.6)	-0.037 (-0.078, 0.003)	0.07	-0.038 (-0.078, 0.003)	0.07
**Learning/Working Memory (n=12,263)**					
No PTSD symptoms	4,050 (33.0)	Ref	Ref	Ref	Ref
1-3 PTSD symptoms	5,054 (41.2)	-0.010 (-0.032, 0.013)	0.39	-0.010 (-0.032, 0.013)	0.40
4-5 PTSD symptoms	2,107 (17.2)	-0.030 (-0.058, -0.002)	0.04	-0.030 (-0.058, -0.001)	0.04
6-7 PTSD symptoms	1,052 (8.6)	-0.066 (-0.102, -0.030)	<0.001	-0.066 (-0.102, -0.031)	<0.001

[a] Beta coefficients of the time x PTSD interaction terms from the linear mixed effect models are shown, representing the difference in one-year change in Cogstate composite scores compared to women with no PTSD symptoms. Practice effects were captured by a categorical variable indicating number of prior tests.
For women with no PTSD symptoms, the estimated rate of decline was -0.065 (95%CI: -0.106 to -0.025) standard deviation (SD)/year in psychomotor speed/attention, and -0.072 (95%CI: -0.108 to -0.035) SD/year in learning/working memory, adjusted for demographic factors and number of prior tests.
Model 1: age at baseline cognitive assessment, race/ethnicity, parental education, participant education, number of prior tests
Model 2: Model 1 + body mass index, smoking status, alcohol use, physical activity, diet quality

© 2022 Roberts AL et al. *JAMA Network Open*.

eTable 7. Association Between PTSD and Rate of Change in Cogstate Composite Scores Using Data Up to the Second, Third, and Fourth Session

	Up to the 2nd session		Up to the 3rd session		Up to the 4th session	
	b **(95% CI)**[a]	***p***	***b*** **(95% CI)**[a]	***p***	***b*** **(95% CI)**[a]	***p***
**Psychomotor Speed/Attention (n=12,248)**						
No PTSD symptoms	Ref	Ref	Ref	Ref	Ref	Ref
1-3 PTSD symptoms	-0.041 (-0.085, 0.003)	0.07	-0.012 (-0.041, 0.017)	0.42	-0.015 (-0.042, 0.011)	0.25
4-5 PTSD symptoms	-0.010 (-0.066, 0.047)	0.73	0.021 (-0.016, 0.058)	0.26	0.015 (-0.018, 0.049)	0.37
6-7 PTSD symptoms	-0.079 (-0.149, -0.008)	0.03	-0.028 (-0.074, 0.019)	0.24	-0.036 (-0.078, 0.006)	0.10
**Learning/Working Memory (n=12,263)**						
No PTSD symptoms	Ref	Ref	Ref	Ref	Ref	Ref
1-3 PTSD symptoms	-0.016 (-0.056, 0.023)	0.41	-0.022 (-0.049, 0.005)	0.10	-0.016 (-0.040, 0.008)	0.19
4-5 PTSD symptoms	-0.045 (-0.095, 0.005)	0.08	-0.041 (-0.075, -0.007)	0.02	-0.040 (-0.070, -0.009)	0.01
6-7 PTSD symptoms	-0.103 (-0.165, -0.041)	0.001	-0.071 (-0.113, -0.028)	0.001	-0.078 (-0.116, -0.040)	<0.001

[a] Beta coefficients of the time x PTSD interaction terms from the linear mixed effect models are shown, representing the difference in one-year change in Cogstate composite scores compared to women with no PTSD symptoms.
Models were adjusted for age at baseline cognitive assessment, race/ethnicity, parental education, participant education.
Sample sizes for Cogstate composite scores by PTSD status were the same as in Table 2.

© 2022 Roberts AL et al. *JAMA Network Open*.

eFigure 1. Flowchart of Analytic Sample Selection

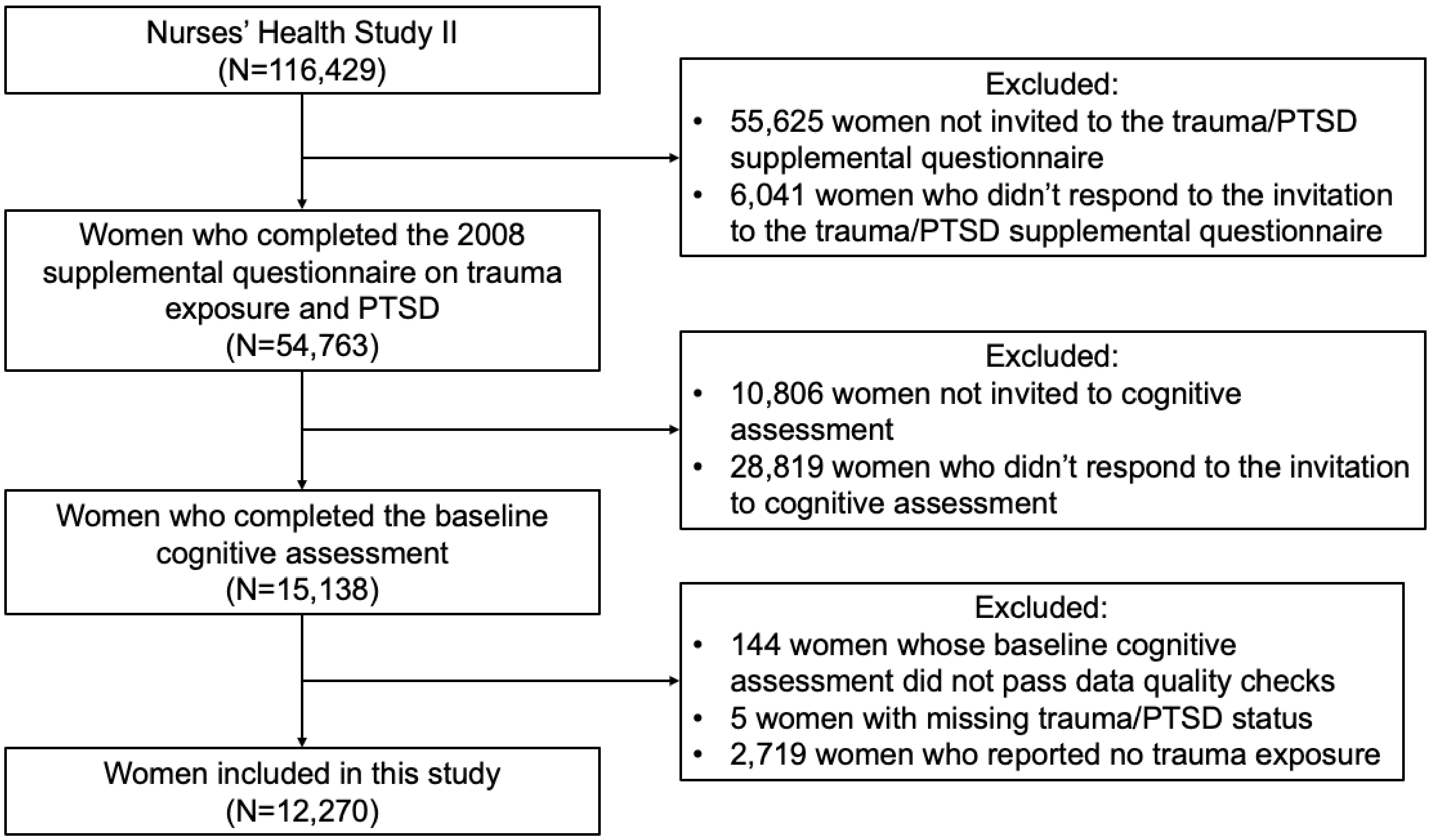

© 2022 Roberts AL et al. *JAMA Network Open*.

eFigure 2. Conceptual Models of PTSD, Cognitive Decline, Depression, and Covariates

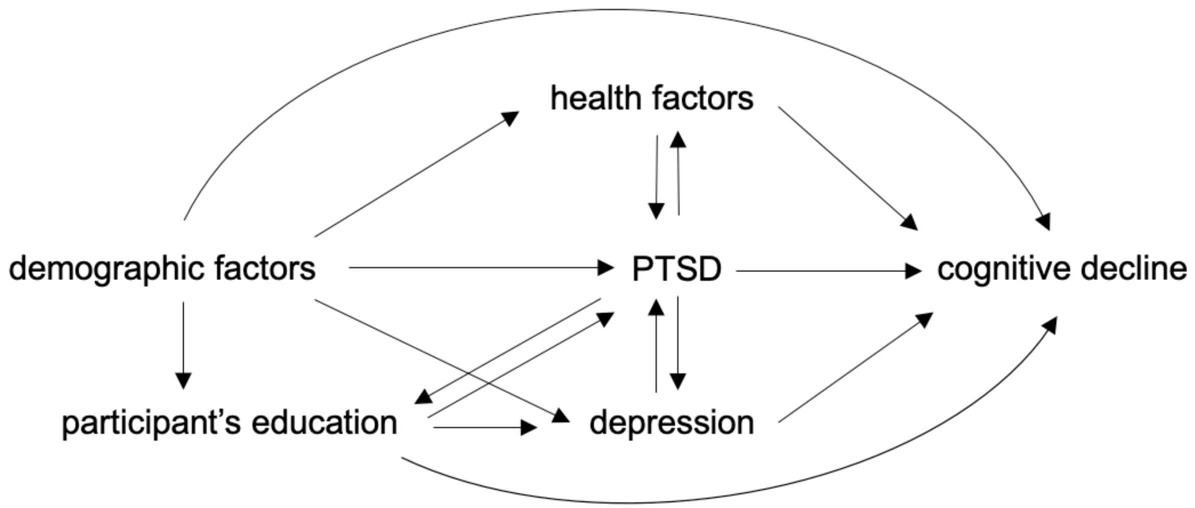

Demographic factors include age, race, parental education. Health factors include body mass index, physical activity, cigarette smoking, alcohol consumption, diet quality, type 2 diabetes, stroke, and myocardial infarction.

© 2022 Roberts AL et al. *JAMA Network Open*.
